# Diagnostic branched tree as an assessment and feedback tool in undergraduate pharmacology education

**DOI:** 10.1186/s12909-023-04342-w

**Published:** 2023-05-24

**Authors:** Ender Tekeş, Çetin Toraman

**Affiliations:** 1 Faculty of Medicine, Department of Pharmacology, Çanakkale Onsekiz Mart University, Çanakkale, 17020 Turkey; 2 Department of Medical Education, Çanakkale Onsekiz Mart University, Çanakkale, Turkey

**Keywords:** Medical Education, Formative Assessment, Diagnostic branched tree, Effective feedback

## Abstract

**Background:**

Multiple-choice, true-false, completion, matching, oral presentation type questions have been used as an evaluation criterion in medical education for many years. Although not as old as other question types, performance evaluation and portfolio-like assessment types, can be called alternative evaluation, have been used for a considerable time. While summative assessment maintains its importance in medical education, the value of formative assessment is gradually increasing. In this research, the use of Diagnostic Branched Tree (DBT), which is used both as a diagnostic and feedback tool, in pharmacology education was examined.

**Methods:**

The study was conducted on 165 students (112 DBT, 53 non-DBT) on the 3rd year of undergraduate medical education. 16 DBTs prepared by the researchers were used as data collection tool. Year 3 first committee was elected for implementation. DBTs were prepared according to the pharmacology learning objectives within the committee. Descriptive statistics, correlation and comparison analyzes were used in the analysis of the data.

**Results:**

DBTs with the most wrong exits are DBTs entitled phase studies, metabolism, types of antagonism, dose-response relationship, affinity and intrinsic activity, G-protein coupled receptors, receptor types, penicillins and cephalosporins. When each question in the DBTs is examined separately, it is seen that most of the students could not answer the questions correctly regarding phase studies, drugs that cause cytochrome enzyme inhibition, elimination kinetics, chemical antagonism definition, gradual and quantal dose response curves, intrinsic activity and inverse agonist definitions, important characteristics of endogenous ligands, changes in the cell as a result of G-protein activation, ionotropic receptor examples, mechanism of action of beta-lactamase inhibitors, excretion mechanism of penicillins, differences of cephalosporins according to generations. As a result of the correlation analysis, the correlation value calculated between the DBT total score and the pharmacology total score in the committee exam. The comparisons showed that the average score of the pharmacology questions in the committee exam of the students who participated in the DBT activity was higher than the students who did not participate.

**Conclusions:**

The study concluded that DBTs are a candidate for an effective diagnostic and feedback tool. Although this result was supported by research at different educational levels, support could not be shown in medical education due to the lack of DBT research in medical education. Future research on DBTs in medical education may strengthen or refute our research results. In our study, receiving feedback with DBT had a positive effect on the success of the pharmacology education.

**Supplementary Information:**

The online version contains supplementary material available at 10.1186/s12909-023-04342-w.

## Background

Multiple choice questions, short answer questions, conventional and structured oral questions are used as traditional assessment and evaluation tools in undergraduate medical education [1]. Recently, alternative measurement and evaluation methods have been started to be used to measure the student’s level of knowledge [2, 3]. Although the validity and reliability of traditional measurement and evaluation methods are high, they are occasionally insufficient in determining learning levels [4]. Therefore, it is important to reach a holistic result in measurement and evaluation by using alternative methods in medical education.

The Diagnostic Branched Tree (DBT) method, which is one of the alternative measurement and evaluation methods, consists of consecutive true and false questions. It aims to detect information patterns and misconceptions in students’ thinking structures (5). The diagnostic tree was defined as a diagnostic alternative evaluation method in 1994 [6] and it was stated that this method would clearly reveal important assumptions regarding knowledge structures (7). While DBT evaluates concept definitions and basic concepts at the initiation steps, it detects misconceptions and their causes at the following steps. The difference of DBT from the classic true-false questions is that the questions are related to each other, and each decision made by the student affects the subsequent decision [8]. Preparing to question the interconnected information network is one of the most important differences compared to the classical true-false tests [5, 7]. However, more effort and experience are required in order to prepare interconnected questions, and they are structured in a more difficult way. Consequently, it is not a widely used method because it is difficult to prepare for inexperienced instructors and the awareness of the method is low among educators.

Although there is no scientific study in which DBT is used in medical school education (for two examples of those used in this study see Appendix 1), studies have been conducted on the primary and secondary education periods. Even though positive data have been obtained about the DBT application in these studies [9, 10], the available data are quite limited.

In the studies in which primary school teachers were included, it was stated that the DBT is among the least known alternative assessment methods, and therefore this method is used less frequently than other alternative methods [11, 12]. In a study conducted among sixth and seventh grade social studies teachers, it was also shown that the DBT is among the least known methods [13]. In addition, it has been determined that science and technology teachers have knowledge deficiencies related to the DBT method [14]. It has been shown that the use of structured grid, DBT and prediction observation explanation tools, which are alternative measurement and evaluation tools, makes a significant difference in the achievement and attitudes of sixth grade students [15]. In a study comparing the DBT method and other alternative methods in seventh grade students, it was determined that the lowest exam score was obtained in the DBT method, and the importance of giving information to increase the level of success was emphasized [4].

Measurement and evaluation in education are used for many purposes. These purposes include establishing classroom equilibrium, planning, and conducting instruction, placing students, providing feedback and incentives, diagnosing student problems and disabilities, and judging and grading academic learning and progress [16, 17]. This purpose can be summative and/or formative. Summative means that the assessment has been conducted for decision-making or certification purposes, such as deciding who is admitted, progresses, or qualifies. Formative relates to the feedback function of assessment or, more precisely, how the assessment informs the students about their performance [18]. According to some authors in the literature, class evaluation serves purposes such as preliminary, formative, summative, and diagnostic [19]. The primary aim is to recognize students’ personal characteristics such as skills, attitudes, and physical features. Formative purpose takes place in the learning-teaching process. It gives information on how far the objectives have been achieved and what kind of adjustments should be made in the learning materials and environment. The summative purpose is used to confirm student achievement and summarize the outcome of the educational process. Certification of training is also within the scope of this purpose. The diagnostic objective is related to the skills and other characteristics that are prerequisites for the current teaching or that ensure the achievement of teaching goals. This assessment attempts to predict the conditions that will negatively affect learning. The DBT method is one of the formative measurement and evaluation methods [20]. According to Miller, Linn and Gronlund [16], diagnostic trees are especially effective in determining what kind of learning difficulties students can experience.

DBTs are used as a feedback tool within the scope of both diagnostic and formative assessment activities in measurement and evaluation. Although there are limited data on the pre-university education period, there is no study comparing DBT with traditional assessment and evaluation methods in medical education. The aim of this research is to use DBT as a diagnostic and feedback tool within the scope of medical school pharmacology course and to examine its effectiveness.

## Methods

The current study utilizes the DBT to determine which subjects were the most incorrectly learned by the students during the teaching process. In this respect, this examination is a descriptive research [21]. On the other hand, in this study, DBTs were used as a feedback tool within the scope of the formative assessment. The effectiveness of the feedback was evaluated by examining the relationships between the pharmacology scores of the exam held at the end of the committee and the results of the DBT. Hence, this study is also a correlational research [22].

### Participants

The participants of the research are the 3rd year students studying at Çanakkale Onsekiz Mart University Faculty of Medicine in the 2022–2023 academic year. The total number of third year students is 165. The research was intended to be carried out with all the students and a meeting was held. Students were informed by the researchers about the purpose of the research and how to implement the application. In addition, it was stated that the participation would be carried out on a voluntary basis and the results of the activity would not have any effect on the success of the students. The number of students participating in the research on a voluntary basis was 112. 53 students did not participate in the study. For this reason, the sampling taken in the research has become convenient sampling (convenience sampling [also known as haphazard or accidental samples.]). Researchers sometimes choose to sample individuals who are already there for research. For example, many researchers collect data from university students because they are ready and willing to participate in research [23]. In addition, we would like to point out that randomization could not be performed because the participation in our study was on a voluntary basis, and this is a limitation of our study in terms of participation and selection bias. The distribution of the students participating in the study according to some of their variables is given in Table 1.

**Table 1:** Distribution of the students participating in the research according to some variables

Variables		N	Percent
Gender	Female	62	55.4
	Male	50	44.6
High School Type	Science High School	55	49.1
	Anatolian High School	50	44.6
	Others	7	6.3
Reason for Choosing Medical School	Because it’s my ideal job	58	51.8
	Because it’s my family’s ideal job	12	10.7
	Because my exam score was matching this school	29	25.9
	Because of prestige and dignity in the society	13	11.6
Does he/she consider choosing Pharmacology for post-graduate education?	Yes	27	24.1
	No	85	75.9
Does he/she read Pharmacology Textbooks?	Yes	40	35.7
	No	72	64.3
Does he/she have the ability to read Pharmacology Textbooks in English?	Yes	30	26.8
	No	82	73.2
What is the importance of pharmacology in medical education?	It is one of the most important lessons	15	13.4
	It is an indispensable course for practicing medicine	45	41.1
	It is a lesson that must be learned to be able to successfully apply other areas of medicine	31	27.7
	It is a difficult lesson and with considerable memorization	11	9.8
	It is a lesson learned only to pass the lesson and be forgotten	9	8
**Total**		112	100

### Data Collection Tool

DBTs were used as a data collection tool in the research. DBTs suitable for the purpose of the research were not found in the literature. For this reason, diagnostic trees have been developed by researchers specifically for the pharmacology course and in accordance with the learning objectives of the third year committee 1. The following steps were followed in the development of the data collection tool:

- The class and time in which DBTs can be used were selected.

The third year of the pharmacology course is the most intensive year at Çanakkale Onsekiz Mart University Faculty of Medicine. Committee 1 was deemed suitable for implementation.

- It was determined for which learning objectives the DBT would be prepared.

DBTs are prepared in accordance with the learning objectives of the pharmacology courses in the 3rd Year Committee 1 curriculum.

- DBTs were prepared for the learning objectives, considering the lecture formats.

It was deemed appropriate to prepare 16 DBTs by examining the learning objectives and the way they were acquired within the subjects. The prepared DBTs were presented to the expert opinion of 4 academicians who are experts in the field of pharmacology and an academician who is an expert in the field of medical education (especially in assessment). Experts were allowed to give expert opinion on each DBT as “appropriate”, “can be used after correction” and “not suitable” (with recommendations if any). Are the expert opinions consistent and reliable? In order to answer this question, 5 experts (4 pharmacology and 1 medical education) using 3 different categories (appropriate, can be used after correction and not suitable) were evaluated with the Krippendorff Alpha coefficient. Inter-rater reliability can be examined with Kripphendorff Alpha in the process performed by more than two raters assigning codes to more than two categories [24]. The calculated value was determined as 0.87. This value is an indicator of high consistency. Upon this, necessary corrections were made on the DBTs in line with the expert opinions. In addition, item-content validity index (I-CVI) and scale-level-content validity index (S-CVI) values were calculated in line with expert opinions. The calculated I-CVI values for the items are not below 0.80. The S-CVI value calculated for the overall measurement tool was also determined as 0.83. According to the literature, these values indicate high content validity [25, 26]. Finally, DBTs were shown to a linguist, and the opinions were taken in terms of clarity, simplicity, and conformity with language rules. According to the opinions received from the linguistics expert, final arrangements were made and the DBTs were finalized before the application (see Appendix 1).

- Pre-application was made to determine the clarity of DBTs before the actual application.

Before the actual application, a preliminary application was made with 5 students to determine the clarity of DBTs. The students with the pre-application are year 4 students and they are the students who took the pharmacology course in the previous year. In the application, the intelligibility of DBTs was found sufficient by 5 students. As a result of all these processes, it was decided by the researchers that the DBTs were ready for the actual application.

- Performing the DBTs actual application.

After the courses of the pharmacology in Committee 1 were completed, the activity was conducted with 112 students who volunteered to participate a week before the committee exam was held. At the end of the activity, the questions in the DBTs were discussed with the students and feedback was given. According to the exits reached by the students, the points with information deficiencies were conveyed to the students.

- After the application, item difficulty, item discrimination and Kuder Richardson (KR-20) reliability analysis of 16 different DBTs and 4 true-false questions in each of the DBTs.

After the application using DBTs, item difficulty, item discrimination evaluation was performed for 4 true-false questions in each of 16 DBTs and a total of 64 true-false questions (see Appendix 2). When the item difficulty value approaches 1, the item is interpreted as an easy question to answer, and when it approaches 0, it is interpreted as a difficult question to answer [27]. As will be seen in Appendix 2, items were generally easy questions. Compared to the other questions, DBT5_4, DBT8_1, DBT8_3, DBT9_2 and DBT15_4 were challenging for the participants. The average test difficulty value of the DBT measurement tool was calculated and a value of 0.77 was obtained. In this case, it can be interpreted that the measurement tool is an easily answerable tool. If the measurement tool developed in the measurement and evaluation literature is to be used for a special purpose and there is no level of ease or difficulty required for this purpose, the items in the measurement tool are expected to be at medium difficulty level (0.50). In this research, the purpose of using DBT is to perform an information-based diagnosis and to give feedback to the students through DBT rather than to determine the learning levels of the students. For this reason, it is expected that the true-false questions in the DBTs should be structured in a way that can be answered easily. In addition, each true-false question is a type of question that offers 50% chance of being answered correctly and has a disadvantage in this respect. When the item discrimination value approaches 1, the item discrimination feature increases, and when it approaches 0 it decreases [27]. In fact, if the item discrimination value is calculated as a negative value, there will be a situation where the item works backwards, with participants with generally low knowledge answering the question correctly, while those with high knowledge answering the question incorrectly. This is an undesirable situation. As seen in Appendix 2, items across DBT mostly had low discrimination value. This situation is correlated with item difficulty, and it was not a surprise for researchers to achieve such a result. The KR-20 reliability level was calculated by using the total scores from all 16 DBTs. The KR-20 reliability level was calculated as 0.77. According to Nunnally and Bernstein [28], sufficient reliability should be at least 0.70 and above. The level of reliability obtained is at an acceptable level according to the literature.

### Data analysis

After the DBT activity, a discussion session was held with the students on the questions and feedback was given. The data obtained in this research and their analyses can be listed as follows:

- Various sociodemographic data: These data were obtained with a form given to students just before the DBT activity. In this form, the students are asked “Gender”, “High School Type”, “Reason for Choosing Medical School”, “Does he/she consider choosing Pharmacology for post-graduate education?”, “Does he/she read Pharmacology Textbooks?”, Does he/she have the ability to read Pharmacology Textbooks in English?” and “What is the importance of pharmacology in medical education?“ data has been collected about the characteristics. These data are summarized with descriptive statistics (frequency and percentages).
- DBT data: The results of the 16 DBTs applied were used in two ways. In each DBT, the students’ answers were given a score of “1” in case of reaching the correct exit and “0” in case of reaching all other exits, and the students’ scores were determined based on a total of 16 points by collecting the correct answers of each student and entering them into the statistical software separately. These data were used for comparison and correlation analyses.
- The scores of the students from pharmacology questions of the 2022–2023 third year first committee exam have been determined. The relationships of these scores with the DBT scores were examined. Moreover, committee exam pharmacology scores of the students who participated and did not participate in the DBT activity were compared.

It was determined that the DBT total score and the committee exam pharmacology total score did not show normal distribution. For this reason, Spearman Brown rank difference correlation coefficient was preferred in the relationship analysis and Mann Whitney U Test was preferred in the comparison analysis.

## Results

### DBT results as a diagnostic tool

One of the expectations in the DBT application is to identify the information that students most misunderstand within the scope of their learning objectives. For this purpose, the status of each student going to the correct exit in each DBT was examined. DBTs with the most wrong exits and the common mistakes made by the students were examined in detail. The correct answer percentages of the students within the scope of DBTs are given in Table 2.

**Table 2:** Percentage of students reaching correct and incorrect exits for each DBT

DBT No	DBT Related Topic	Correct (%)	Incorrect (%)	DBT No	DBT Related Topic	Correct (%)	Incorrect (%)
DBT1	Phase studies	24(21.4)	88(78.6)	DBT9	Affinity and intrinsic activity	26(23.2)	86(76.8)
DBT2	Sources and nomenclature of drugs	82(73.2)	30(26.8)	DBT10	Ligands and spare receptors	47(42)	65(58)
DBT3	Absorption	76(67.9)	36(32.1)	DBT11	G-protein coupled receptors	34(30.4)	78(69.6)
DBT4	Distribution	55(49.1)	57(50.9)	DBT12	Receptor types	34(30.4)	78(69.6)
DBT5	Metabolism	30(26.8)	82(73.2)	DBT13	Penicillins-1	72(64.3)	40(35.7)
DBT6	Elimination	48(42.9)	64(57.1)	DBT14	Penicillins-2	45(40.2)	67(59.8)
DBT7	Types of antagonism	41(36.6)	71(63.4)	DBT15	Penicillins-3	32(28.6)	80(71.4)
DBT8	Dose-response relationship	5(4.5)	107(95.5)	DBT16	Cephalosporins	37(33)	75(67)

Among the DBTs, the most accurate exits are reached in DBTs entitled sources and nomenclature of drugs, absorption and penicillins-1. DBTs with half the percentages of reaching the correct exit and reaching the wrong exit are DBTs entitled distribution, elimination, ligands and spare receptors and penicillins-2. DBTs with the most wrong exits are DBTs entitled phase studies, metabolism, types of antagonism, dose-response relationship, affinity and intrinsic activity, G-protein coupled receptors, receptor types, penicillins-3 and cephalosporins. On the DBTs, in which a large number of students reached the wrong exit, analyzes were made on the basis of the wrong answers of the students for each question. The results of the analysis are given in Table 3.

**Table 3:** The most incorrectly answered questions in DBTs with the most mistakes done

DBT No	Question No	Incorrect Responses (%)	Question
DBT1	1	46 (41.1)	Phase 1 studies are mainly efficacy studies
	2	8 (7.1)	A drug candidate can be directly investigated in human studies without testing in animals
	3	58 (51.8)	The LD50 is detected in phase 2 studies
	4	15 (13.4)	If the Phase 4 study is successful, a drug license application is made
DBT5	1	33 (29.5)	The purpose of drug metabolism is to make the drug more lipophilic
	2	35 (31.3)	The oxidation carried out by microsomal enzymes in the liver is the second phase reaction
	3	13 (11.6)	The cytochrome P450 enzyme that metabolizes 50% of all drugs is CYP3A4/5
	4	69 (61.6)	Chronic alcohol intake causes CYP enzyme inhibition
DBT6	1	3 (2.7)	Most of the drugs are eliminated by first order kinetics
	2	37 (33.0)	The elimination rate of drugs eliminated with first order kinetics is independent of the plasma concentration
	3	32 (28.6)	In the first order kinetics, a constant amount of the drug is eliminated per unit of time
	4	25 (22.3)	In the zero-order kinetics, the half-life remains constant
DBT7	1	52 (46.4)	The inactivation of an acidic drug with a basic drug is pharmacological antagonism
	2	6 (5.4)	The type of antagonism seen between a vasoconstrictor drug and a vasodilator drug is physiological antagonism
	3	17 (15.2)	The main effect of the non-competitive antagonist is to reduce the efficacy of the agonist
	4	31 (27.7)	A competitive antagonist does not alter the efficacy of the agonist, but increases its potency
DBT8	1	84 (75.0)	A plasma concentration time curve is drawn to demonstrate the potency of an agonist drug
	2	31 (27.68)	The smaller the EC50 of a drug, the higher its efficacy
	3	85 (75.9)	A gradual dose response curve is used when the drug effect is all or none
	4	8 (7.14)	The greater the therapeutic index value of a drug, the safer it is
DBT9	1	50 (44.6)	The ability to activate the receptor after binding to the receptor is called affinity
	2	74 (66.1)	An antagonist is a drug that affects in the opposite direction by binding to the receptor
	3	7 (6.3)	A partial agonist is a drug that has an agonist-like effect if used alone and reduces the agonist’s effect if used together with an agonist
	4	21 (18.8)	The drug with negative intrinsic activity is an inverse agonist
DBT10	1	17 (15.2)	If an agonist has produced maximum effect, it always means that it binds to all receptors
	2	0 (0)	The substance that selectively binds to a receptor is called ligand
	3	4 (3.6)	In the absence of spare receptors, the concentration that produces half the maximum effect is equal to the concentration required to bind half of all receptors
	4	54 (48.2)	Endogenous ligands are always agonists
DBT11	1	14 (12.5)	No secondary messenger is used in G-protein coupled receptor-mediated signaling
	2	60 (53.6)	Gs type G-protein activates phospholipase C
	3	19 (16.7)	Gq type G-protein activation causes an increase in intracellular calcium
	4	24 (21.4)	Bronchodilation is seen as a result of adenylate cyclase activation and cyclic AMP increase with a Gs type G-protein mediated effect
DBT12	1	25 (22.3)	Adrenergic and muscarinic receptors are G-protein coupled receptors
	2	51 (45.5)	Nicotinic receptors are G-protein coupled receptors
	3	12 (10.7)	The insulin receptor is a tyrosine kinase receptor
	4	19 (17.0)	Thyroid hormone receptor is in the nucleus
DBT14	1	9 (8.0)	Beta lactam antibiotics are safe during pregnancy
	2	15 (13.4)	Piperacillin is administered only orally
	3	57 (50.9)	Sulbactam inhibits transpeptidase enzyme
	4	7 (6.3)	Aztreonam can be used in patients with penicillin allergy
DBT15	1	12 (10.7)	Penicillins are bacteriostatic
	2	38 (33.9)	Penicillins reversibly inhibit the transpeptidase enzyme
	3	4 (3.6)	The main mechanism of penicillin resistance is the production of beta-lactamase enzyme by the bacteria
	4	66 (58.9)	Excretion of penicillins occurs by glomerular filtration
DBT16	1	27 (24.1)	Ceftaroline is the only effective cephalosporin for MRSA
	2	62 (55.4)	Cefepime can cross the blood brain barrier but is unstable to beta-lactamase
	3	12 (10.7)	Ceftazidime is effective against Pseudomonas aeruginosa
	4	4 (3.6)	Cefazolin is the most commonly used cephalosporin in surgical prophylaxis

When each question in the DBTs is examined separately, it is seen that most of the students could not answer the questions correctly regarding phase studies, drugs that cause cytochrome enzyme inhibition, elimination kinetics, chemical antagonism definition, gradual and quantal dose response curves, intrinsic activity and inverse agonist definitions, important characteristics of endogenous ligands, changes in the cell as a result of G-protein activation, ionotropic receptor examples, mechanism of action of beta-lactamase inhibitors, excretion mechanism of penicillins, differences of cephalosporins according to generations. The questions prepared include basic pharmacology topics such as pharmacokinetics and pharmacodynamics, and antibiotics such as penicillins and cephalosporins.

### Usefulness of DBT as a feedback tool

A total of 112 students participated in the DBT study. The relationship between the total scores obtained from the DBTs and the total scores they obtained from the pharmacology questions in the committee exam was examined. The results are given in Table 4.

**Table 4:** The relationship between DBT scores and committee exam pharmacology scores

Variables	N	Spearman r	p
DBT Total Score * Total Score of Pharmacology Questions in the Committee Exam	112	0.446	< 0.0001

As a result of the correlation analysis, the correlation value calculated between the DBT total score and the pharmacology total score in the committee exam was 0.446. The obtained correlation value is a moderate correlation indicator [29–32]. The calculated correlation is positive and significant. In this case, it can be interpreted that when the DBT total score increases, the pharmacology total score in the committee exam also increases.

While 112 students participated in the DBT activity, 53 students did not volunteer and did not participate. Comparison of the total score of the pharmacology questions in the committee exam of the students who did and did not participate DBT activity was performed. The results are shown in Table 5.

**Table 5:** Comparison of committee exam pharmacology scores according to DBT activity participation

Groups	N	Mean (S. Deviation)	Median (Min.-Max.)	U Test	p	Effect Size (Rank Biserial Correlation)
DBT Groups	112	33.1(5.5)	34(17–44)	1725	< 0.00001	0.419
Non-DBT Groups	53	28(7.3)	29(10–43)			

The comparisons showed that the average of the total score of the pharmacology questions in the committee exam of the students who participated in the DBT activity was higher than the students who did not participate. The significant difference obtained was recorded in the medium effect size [33]. This situation may be related to the fact that the students participating in the DBT activity are students who take care of their participation in the lessons, as well as it can be interpreted that they benefit from receiving feedback with the DBT activity.

## Discussion

Formative assessment and evaluation methods, which make the measurement and evaluation methods used in undergraduate medical education from being monotonous, provide feedback to the students while the education continues, and thus enable the students to be aware of their deficiencies, are being added to the medical school education curricula day by day. Summative assessments are designed to ensure the self-regulation and accountability of universities, while formative assessments aim to guide the learning process by focusing on providing feedback to students. Formative assessments such as reflective portfolios, self and peer assessment, mini-clinical evaluation exercise and objective structured clinical examination stations are used in medical education programs [34].

The DBT method, which is an alternative formative method, is an application consisting of consecutive true and false questions, in which the student will take two different paths according to the choice of true or false for each question, and can reach a total of 4, 8, 16 or 32 different exits depending on the number of columns. The exit gate that the student reaches in the last question gives information about which questions were answered incorrectly. Thus, it is very practical in terms of showing the student’s lack of knowledge and allows special feedback to be given to the student. In addition, when the data of all students are examined, it provides the instructor with data about the general knowledge deficiencies of the class by showing most frequently incorrectly answered questions in the whole group. Its designation as a diagnostic is based on the fact that this method quickly distinguishes errors on a broad, branched pathway. For this reason, this method not only makes measurement and evaluation, but also detects the subjects that are difficult, not well understood, and unlearned on a student-based or class-based basis. The data obtained in this method will also allow the trainer to update the training materials that will be used in the following years and to make them more understandable.

Although there are data on the fact that formative applications improve the learning process [35–37] in studies comparing formative assessment with summative assessment in medical school students, there is no data on the use of DBT method in medical school education programs. In our study, students who participated in the DBT activity had higher scores in the pharmacology questions of the committee exam than those who did not participate in the activity.

The available DBT data are the data about its use in the pre-university education period. A web-based application inspired by the descriptive branched tree application significantly increased the science and technology course scores of sixth, seventh and eighth grade students compared to traditional education [10]. In a study conducted in elementary school eighth grade students, it was concluded that the DBT technique, which is used by integrating conceptual change into the text, is effective in correcting misconceptions [9]. It has been determined that the DBT developed for the eleventh-grade high school chemistry course is reliable in terms of difficulty and distinctiveness [38].

The DBT method has some difficulties and disadvantages. It is believed that the reliability of the scores obtained by this method is not high due to the high factor of chance in answering correctly [39]. The chance of answering each true-false question in the flow is 50%. Although this statement is correct when evaluated step by step, if the tree is evaluated as a whole, that is, if only reaching the correct exit is a success criterion, the probability of correct prediction is lower than other techniques [8]. For example, while this ratio is 1/5 in a five-choice multiple choice question, this ratio is 1/8 in a DBT with 8-exit and 1/16 in a DBT with 16-exit. However, DBT may be an inadequate method for measuring high-level learning skills due to the fact that it consists of true-false questions [8]. In addition, it is thought to be a more appropriate method for small groups, as it will be more difficult to give student-based feedback to a large group of students. Moreover, it was stated as a difficulty that the students could not see all of the questions in this method [5]. For example, DBT studies conducted among high school students [38, 40, 41] and education faculty university students [42] have different statements in each cell. However, when this type of DBT is used, it is not possible for every student to see all of the questions since the path to be taken is different according to the answer given by the student. Therefore, in our study, we used the same questions in the same column so that each question could be seen by all students. Thus, we aimed to reduce the deviations due to answering different questions. In this way, our other purpose in practice was to see in which parts students and the class had difficulties and to give appropriate feedback. We first determined the main topics in accordance with our medical school curriculum, and then we started to prepare the DBT questions. Since there is no example in the literature for medical school, we thought it would be more appropriate to make some changes in the format used mostly in the pre-university education period. For example, although 3-tier DBT was used in DBT studies conducted with 10th grade [40, 41] and 11th grade high school students [38], we used 4-tier DBT in our study. While a general statement is written in the 1st tier of the DBT concept, that is, the general knowledge level of the student is questioned, more specific statements about the same topic and statements about subtopics are added to the next tiers. Thus, the level of knowledge of the student in the sub-topics of the relevant subject is also evaluated. In fact, the purpose of using 4-tiered DBT instead of 3-tiered structure was to increase the number and variety of sub-topics questioned.

DBT should be done in the classroom in order to detect students’ misconceptions and to show whether the relationships between concepts are established correctly [43]. We conducted our study evaluation face-to-face in a classroom setting.

It has been shown that DBT method is not preferred by pre-university social science teachers. As the reason, it is stated that there is insufficient competence in the use of this method [20].

Pharmacology courses in undergraduate education programs in medical faculties are held in the third year of education in most universities in the form of classical classroom education, and assessment and evaluation are done in the form of committee exams consisting of multiple-choice questions. Factors such as the quality level and the difficulty level of the multiple-choice questions push the students to think and work on this axis, not to learn the subjects and increase their knowledge, but to pass the class. For students, getting a passing grade is the main goal, and for this, they try to save the day by only studying from the lecture notes, instead of obtaining long-term gains by studying comprehensively from the textbooks. Approximately 2/3 (64.3%) of the students who participated in our study stated that they did not read pharmacology textbooks. If the assessment and evaluation questions are not of high quality, the traditional exams do not go beyond distinguishing those who memorize well and those who do not memorize well.

Due to the reasons mentioned, innovative practices aimed at improving and enriching the measurement and evaluation method in the education system will be valuable. Formative assessments in the form of mid-term exams while training continues are a good example of these innovative practices.

The questions prepared in our study were applied at the beginning of the academic year in accordance with the curriculum, so the topics mainly include pharmacokinetics and pharmacodynamics, which are the basic topics of pharmacology, and where concepts and definitions are more intense. When we examined the most frequently incorrectly answered questions in our study, it was found that more mistakes were made in the questions where information about new concepts, definitions and differentiating points were tested. It is expected that students will make mistakes in this type of basic information in a lesson where they have just started to learn the terminology. It is thought that students who do not repeat and reinforce their knowledge make more mistakes. Furthermore, the majority of the students in the classroom only study from the instructor’s lecture notes and do not read textbooks or other materials related to the course at all. Students had difficulty with questions that were not clearly stated in the lecture notes but could be solved by combining the information from different parts of the course (For example, the 3rd question in the 1st tree). Further studies are required for specific assessments of the systems and diseases.

The DBT application not only makes measurement and evaluation, but also detects the subjects that are difficult, not well understood, and unlearned, on a student-based or class-based basis, in interrelated subjects. Thanks to the feedback given to the student after the activity, students will be able to identify their shortcomings, realize the current level of knowledge and the subtopics that need to be improved. In this method, it will be possible to study more focused and participate in the main exam more prepared.

Using only traditional tests is insufficient for measurement and evaluation [5]. Testing different measurement and evaluation methods in undergraduate pharmacology education and adding them to the curriculum after observing positive results will be beneficial in improving the quality of pharmacology education. In this context, there is a need for new study data in which different evaluation methods are tested in comparison with control groups. It is obvious that making the pharmacology course, which has an important place in undergraduate education, higher quality in terms of both educational materials and assessment and evaluation methods will provide benefits in educating better physicians.

## Electronic supplementary material

Below is the link to the electronic supplementary material.

Appendix 1 Feedbacks. Appendix 2 Item Analysis.

## Data Availability

The datasets used and/or analyzed during the study are available from the corresponding author upon reasonable request.
