# Supplementary material for: Diagnostic branched tree as an assessment and feedback tool in undergraduate pharmacology education
Source: BMC Med Educ. 2023 May 24;23:374. doi: 10.1186/s12909-023-04342-w (PMC10210285; doi:10.1186/s12909-023-04342-w)
Supplement: Supplementary file 1 — Appendix 1 Feedbacks. Appendix 2 Item Analysis. [file 12909_2023_4342_MOESM1_ESM.pdf]

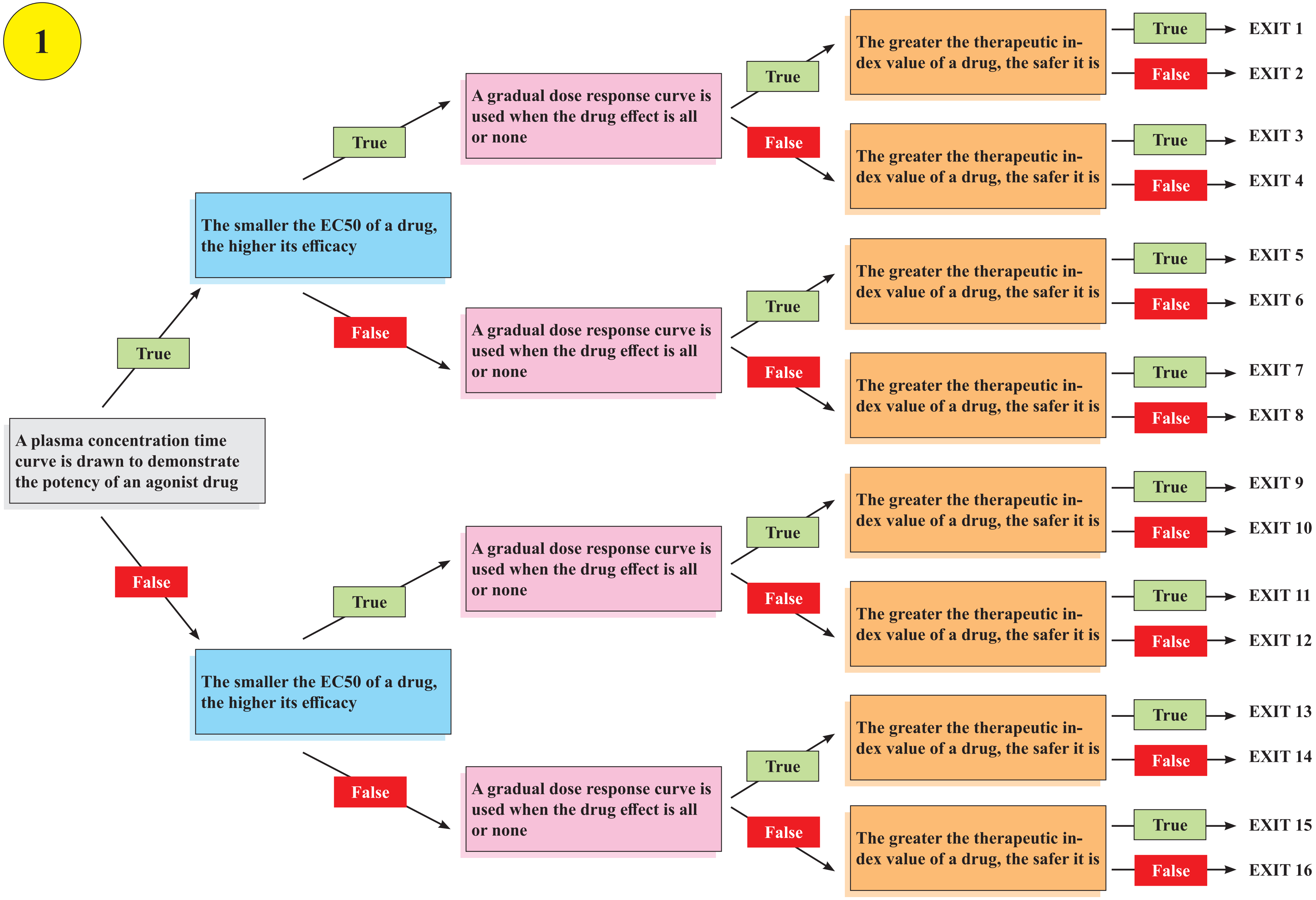
1
A plasma concentration time curve is drawn to demonstrate the potency of an agonist drug
True
The smaller the EC50 of a drug, the higher its efficacy
True
A gradual dose response curve is used when the drug effect is all or none
True
The greater the therapeutic index value of a drug, the safer it is
True
EXIT 1
False
EXIT 2
False
The greater the therapeutic index value of a drug, the safer it is
True
EXIT 3
False
EXIT 4
False
A gradual dose response curve is used when the drug effect is all or none
True
The greater the therapeutic index value of a drug, the safer it is
True
EXIT 5
False
EXIT 6
False
The greater the therapeutic index value of a drug, the safer it is
True
EXIT 7
False
EXIT 8
False
The smaller the EC50 of a drug, the higher its efficacy
True
A gradual dose response curve is used when the drug effect is all or none
True
The greater the therapeutic index value of a drug, the safer it is
True
EXIT 9
False
EXIT 10
False
The greater the therapeutic index value of a drug, the safer it is
True
EXIT 11
False
EXIT 12
False
A gradual dose response curve is used when the drug effect is all or none
True
The greater the therapeutic index value of a drug, the safer it is
True
EXIT 13
False
EXIT 14
False
The greater the therapeutic index value of a drug, the safer it is
True
EXIT 15
False
EXIT 16

Appendix 1 Feedbacks

Exit 1: If you have reached exit 1, you have information deficiencies regarding potency, efficacy and the quantal dose response curve. We recommend that you study the relevant topic/topics again.

Exit 2: If you have reached exit 2, you have information deficiencies regarding potency, efficacy, quantal dose response curve and therapeutic index. We recommend that you study the relevant topic/topics again.

Exit 3: If you have reached exit 3, you have information deficiencies regarding potency and efficacy. We recommend that you study the relevant topic/topics again.

Exit 4: If you have reached exit 4, you have information deficiencies regarding potency, efficacy and therapeutic index. We recommend that you study the relevant topic/topics again.

Exit 5: If you have reached exit 5, you have information deficiencies regarding potency and quantal dose response curve. We recommend that you study the relevant topic/topics again.

Exit 6: If you have reached exit 6, you have information deficiencies regarding potency, quantal dose response curve and therapeutic index. We recommend that you study the relevant topic/topics again.

Exit 7: If you have reached exit 7, you have information deficiencies regarding potency. We recommend that you study the relevant topic/topics again.

Exit 8: If you have reached exit 8, you have information deficiencies regarding potency and therapeutic index. We recommend that you study the relevant topic/topics again.

Exit 9: If you have reached exit 9, you have information deficiencies regarding efficacy and quantal dose response curve. We recommend that you study the relevant topic/topics again.

Exit 10: If you have reached exit 10, you have information deficiencies regarding efficacy, quantal dose response curve and therapeutic index. We recommend that you study the relevant topic/topics again.

Exit 11: If you have reached exit 11, you have information deficiencies regarding efficacy. We recommend that you study the relevant topic/topics again.

Exit 12: If you have reached exit 12, you have information deficiencies regarding efficacy and therapeutic index. We recommend that you study the relevant topic/topics again.

Exit 13: If you have reached exit 13, you have information deficiencies regarding quantal dose response curve. We recommend that you study the relevant topic/topics again.

Exit 14: If you have reached exit 14, you have information deficiencies regarding quantal dose response curve and therapeutic index. We recommend that you study the relevant topic/topics again.

Exit 15: If you have reached exit 15, you have correctly answered all questions on potency, efficacy, quantal dose response curve and therapeutic index. Congratulations!

Exit 16: If you have reached exit 16, you have information deficiencies regarding therapeutic index. We recommend that you study the relevant topic/topics again.

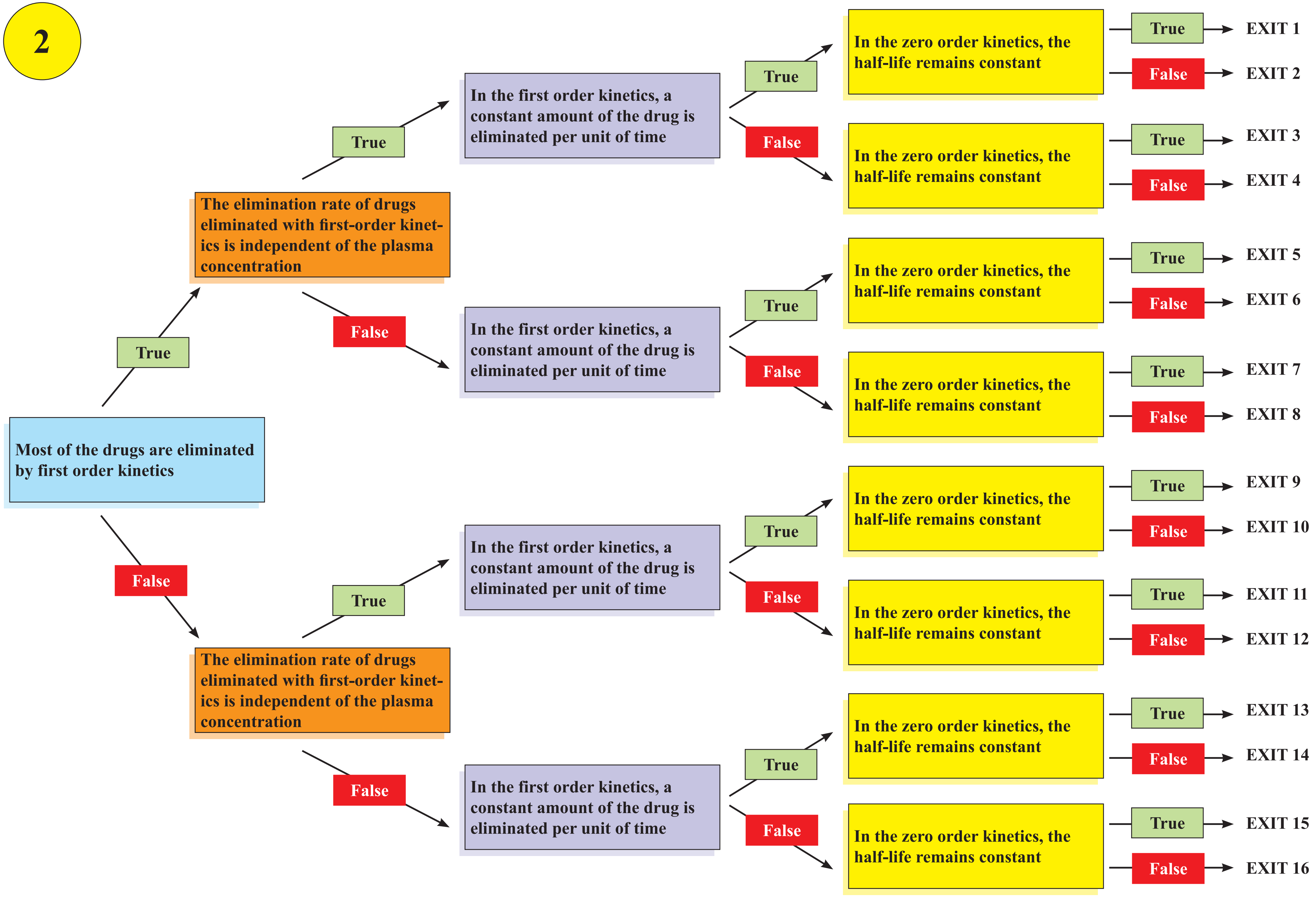
2
Most of the drugs are eliminated by first order kinetics
True
The elimination rate of drugs eliminated with first-order kinetics is independent of the plasma concentration
True
In the first order kinetics, a constant amount of the drug is eliminated per unit of time
True
In the zero order kinetics, the half-life remains constant
True
EXIT 1
False
EXIT 2
False
In the zero order kinetics, the half-life remains constant
True
EXIT 3
False
EXIT 4
False
In the first order kinetics, a constant amount of the drug is eliminated per unit of time
True
In the zero order kinetics, the half-life remains constant
True
EXIT 5
False
EXIT 6
False
In the zero order kinetics, the half-life remains constant
True
EXIT 7
False
EXIT 8
False
The elimination rate of drugs eliminated with first-order kinetics is independent of the plasma concentration
True
In the first order kinetics, a constant amount of the drug is eliminated per unit of time
True
In the zero order kinetics, the half-life remains constant
True
EXIT 9
False
EXIT 10
False
In the zero order kinetics, the half-life remains constant
True
EXIT 11
False
EXIT 12
False
In the first order kinetics, a constant amount of the drug is eliminated per unit of time
True
In the zero order kinetics, the half-life remains constant
True
EXIT 13
False
EXIT 14
False
In the zero order kinetics, the half-life remains constant
True
EXIT 15
False
EXIT 16

Appendix 2 Feedbacks

Exit 1: If you have reached exit 1, you have information deficiencies regarding correlation of elimination rate with concentration, first order elimination kinetics and zero order elimination kinetics. We recommend that you study the relevant topic/topics again.

Exit 2: If you have reached exit 2, you have information deficiencies regarding correlation of elimination rate with concentration and first order elimination kinetics. We recommend that you study the relevant topic/topics again.

Exit 3: If you have reached exit 3, you have information deficiencies regarding correlation of elimination rate with concentration and zero order elimination kinetics. We recommend that you study the relevant topic/topics again.

Exit 4: If you have reached exit 4, you have information deficiencies regarding correlation of elimination rate with concentration. We recommend that you study the relevant topic/topics again.

Exit 5: If you have reached exit 5, you have information deficiencies regarding first order elimination kinetics and zero order elimination kinetics. We recommend that you study the relevant topic/topics again.

Exit 6: If you have reached exit 6, you have information deficiencies regarding first order elimination kinetics. We recommend that you study the relevant topic/topics again.

Exit 7: If you have reached exit 7, you have information deficiencies regarding zero order elimination kinetics. We recommend that you study the relevant topic/topics again.

Exit 8: If you have reached exit 8, you have correctly answered all questions on elimination of drugs, correlation of elimination rate with concentration, first order elimination kinetics and zero order elimination kinetics. Congratulations!

Exit 9: If you have reached exit 9, you have information deficiencies regarding elimination of drugs, correlation of elimination rate with concentration, first order elimination kinetics and zero order elimination kinetics. We recommend that you study the relevant topic/topics again.

Exit 10: If you have reached exit 10, you have information deficiencies regarding elimination of drugs, correlation of elimination rate with concentration and first order elimination kinetics. We recommend that you study the relevant topic/topics again.

Exit 11: If you have reached exit 11, you have information deficiencies regarding elimination of drugs, correlation of elimination rate with concentration and zero order elimination kinetics. We recommend that you study the relevant topic/topics again.

Exit 12: If you have reached exit 12, you have information deficiencies regarding elimination of drugs and correlation of elimination rate with concentration. We recommend that you study the relevant topic/topics again.

Exit 13: If you have reached exit 13, you have information deficiencies regarding elimination of drugs, first order elimination kinetics and zero order elimination kinetics. We recommend that you study the relevant topic/topics again.

Exit 14: If you have reached exit 14, you have information deficiencies regarding elimination of drugs and first order elimination kinetics. We recommend that you study the relevant topic/topics again.

Exit 15: If you have reached exit 15, you have information deficiencies regarding elimination of drugs and zero order elimination kinetics. We recommend that you study the relevant topic/topics again.

Exit 16: If you have reached exit 16, you have information deficiencies regarding elimination of drugs. We recommend that you study the relevant topic/topics again.
